# Trait Rumination Influences Neural Correlates of the Anticipation but Not the Consumption Phase of Reward Processing

**DOI:** 10.3389/fnbeh.2017.00085

**Published:** 2017-05-10

**Authors:** Natália Kocsel, Edina Szabó, Attila Galambos, Andrea Édes, Dorottya Pap, Rebecca Elliott, Lajos R. Kozák, György Bagdy, Gabriella Juhász, Gyöngyi Kökönyei

**Affiliations:** ^1^Doctoral School of Psychology, Eötvös Loránd UniversityBudapest, Hungary; ^2^Institute of Psychology, Eötvös Loránd UniversityBudapest, Hungary; ^3^MTA-SE NAP B Genetic Brain Imaging Migraine Research Group, Hungarian Academy of Sciences, Semmelweis UniversityBudapest, Hungary; ^4^Department of Pharmacodynamics, Faculty of Pharmacy, Semmelweis UniversityBudapest, Hungary; ^5^Neuroscience and Psychiatry Unit, University of ManchesterManchester, UK; ^6^Manchester Academic Health Sciences Centre, University of ManchesterManchester, UK; ^7^MR Research Center, Semmelweis UniversityBudapest, Hungary; ^8^MTA-SE Neuropsychopharmacology and Neurochemistry Research Group, Hungarian Academy of Sciences, Semmelweis UniversityBudapest, Hungary

**Keywords:** rumination, reward, loss, anticipation, consumption, monetary incentive delay task, fMRI

## Abstract

Cumulative evidence suggests that trait rumination can be defined as an abstract information processing mode, which leads people to constantly anticipate the likely impact of present events on future events and experiences. A previous study with remitted depressed patients suggested that enhanced rumination tendencies distort brain mechanisms of anticipatory processes associated with reward and loss cues. In the present study, we explored the impact of trait rumination on neural activity during reward and loss anticipation among never-depressed people. We analyzed the data of 37 healthy controls, who performed the monetary incentive delay (MID) task which was designed for the simultaneous measurement of the anticipation (motivational) and consumption (hedonic) phase of reward processing, during functional magnetic resonance imaging (fMRI). Our results show that rumination—after controlling for age, gender, and current mood—significantly influenced neural responses to reward (win) cues compared to loss cues. Blood-oxygenation-level-dependent (BOLD) activity in the left inferior frontal gyrus (IFG) triangularis, left anterior insula, and left rolandic operculum was positively related to Ruminative Response Scale (RRS) scores. We did not detect any significant rumination-related activations associated with win-neutral or loss-neutral cues and with reward or loss consumption. Our results highlight the influence of trait rumination on reward anticipation in a non-depressed sample. They also suggest that for never-depressed ruminators rewarding cues are more salient than loss cues. BOLD response during reward consumption did not relate to rumination, suggesting that rumination mainly relates to processing of the motivational (wanting) aspect of reward rather than the hedonic (liking) aspect, at least in the absence of pathological mood.

## Introduction

Rumination, according to the widely used Response Style Theory, is a passive and repetitive thinking process, which focuses one's attention to depressive symptoms, and to reasons and consequences of these symptoms (Nolen-Hoeksema, 1991). Many studies in the past decades have demonstrated that the tendency to ruminate reliably predicts the development of depressive symptoms (Sarin et al., 2005), and major depressive disorder (MDD; Nolen-Hoeksema, 2000). However, the latest results suggest that rumination is a transdiagnostic characteristic, which can be present in other psychopathologies, and in addition it can influence the thinking process of healthy (never-depressed) people (Aldao et al., 2010; Nolen-Hoeksema and Watkins, 2011; McLaughlin et al., 2014).

According to recent theories, the primary information processing mode is the key mechanism in the maintenance and escalation of rumination (Watkins et al., 2008). Watkins et al. (2015) suggest that depressive rumination is associated with abstract information processing, which (contrary to concrete processing), generates incomplete mental representations of events from what the contextual details are lacking. For this reason, abstract processing leads to elevated implicational thinking, when people permanently anticipate the likely impact of present experiences on future events (Watkins et al., 2008, 2015).

One of the core aspects of these summarized (and simplified) representations is the emotional charge which, regardless of its valence (positive, negative, or neutral), will strongly influence the estimation processes and leads to emotional extrapolation. This means that ruminators build their future anticipations on the emotional tone of expected future events (Watkins et al., 2015). Ruminative tendencies do not always lead to negative anticipations, but many times impair the information processing, since rumination by definition, exaggerates the importance of negative information and overgeneralizes and amplifies the incidence of casual failures (Van Lier et al., 2014).

Here we hypothesize that *never-depressed trait* ruminators may experience difficulties in the differentiation of important and unimportant failures and in the processing of cues indicating reward or punishment. The findings of Whitmer et al. (2012) provide the rationale for our hypothesis. They found that induced (*state*) rumination in *depressed* participants was associated with generally reduced sensitivity to punishment cues compared to reward cues and reduced sensitivity to the probability that a stimulus would be associated with punishment (Whitmer et al., 2012). Research exploring the neural basis of rumination and reward/punishment signals is very limited. A recent study of patients with remitted depression failed to find any relationship between rumination and brain responses to cues predicting reward and punishment. Instead, an association between trait rumination and punishment consumption was observed, specifically, trait rumination had a strong negative correlation with the activation of superior frontal gyrus during loss outcomes among participants with remitted depression, but not in the control group (Schiller et al., 2013). It remains unclear whether this result could be attributed to never-depressed ruminators.

In fMRI studies, rewarding or punishing situations are often modeled with monetary win and loss games, which reliability have been verified by many previous findings (Knutson et al., 2003, 2008; Bjork et al., 2004). In particular, the commonly used monetary incentive delay (MID) task, has characterized extensively the neural bases of anticipatory and consummator stages of reward and loss processing (Dillon et al., 2008; Pizzagalli et al., 2009). Our aim in the present study was to use the MID to explore the impact of trait rumination on neural activity during reward and loss anticipation and consumption among healthy (never-depressed) people.

## Methods

### Participants

Thirty-seven (15 males, 22 females, mean age ± *SD*: 25.92 ± 4.18) right-handed volunteers were included in the present study. Participants aged between 18 and 38 years were recruited via newspaper and university advertisements. All volunteers were tested for eligibility. Inclusion criteria were right-handedness assessed with a standardized handedness questionnaire (Oldfield, 1971), and normal or corrected to normal vision. Exclusion criteria were any history of medical, neurological or psychiatric disorder diagnosed by senior neurologist and psychiatrist researchers. Individuals with history of psychotropic medication use were also excluded.

This study was carried out in accordance with the recommendations of Scientific and Research Ethics Committee of the Medical Research Council (Hungary) with written informed consent from all subjects. All subjects gave written informed consent in accordance with the Declaration of Helsinki. The protocol was approved by the Scientific and Research Ethics Committee of the Medical Research Council (Hungary).

### Self-report measures

Rumination was assessed by the *Ruminative Response Scale (RRS*) of the widely used Response Style Questionnaire (Nolen-Hoeksema and Morrow, 1993). The original 22-item version of the scale contains items such as “*What am I doing to deserve this?”, “I won't be able to do my job if I don't snap out of this.”* The respondents had to indicate on a 4-point Likert scale how often the items apply to themselves (1, almost never; 4, almost always). The internal consistency of the RRS was good (Cronbach α = 0.89). The total RRS score was used in the correlational analysis.

Depressive symptoms were measured by the validated Hungarian adaptation of the *Zung Self-Rating Depression Scale (ZSDS*; Zung, 1965; Simon, 1998). The ZSDS is a 20-item instrument that quantifies the depressive symptoms via psychological *(“I am more irritable than usual”)*, affective *(“I feel down-hearted and blue”)* and somatic *(“I have trouble sleeping at night”)* dimensions. Participants were asked to score the items on a 1–4 scale (i.e., 1 = a little of the time, 4 = most of the time). Internal consistency of the ZSDS proved to be good (Cronbach α = 0.83). The total ZSDS score was used in the correlational analysis. Besides the RRS and ZSDS the test battery contained basic background questions regarding age, sex, ethnicity, family, and personal psychiatric history.

The self-report measures were taken some days before the scan sessions, in order to avoid any unwanted rumination induction.

### MID task

**Figure 1:**
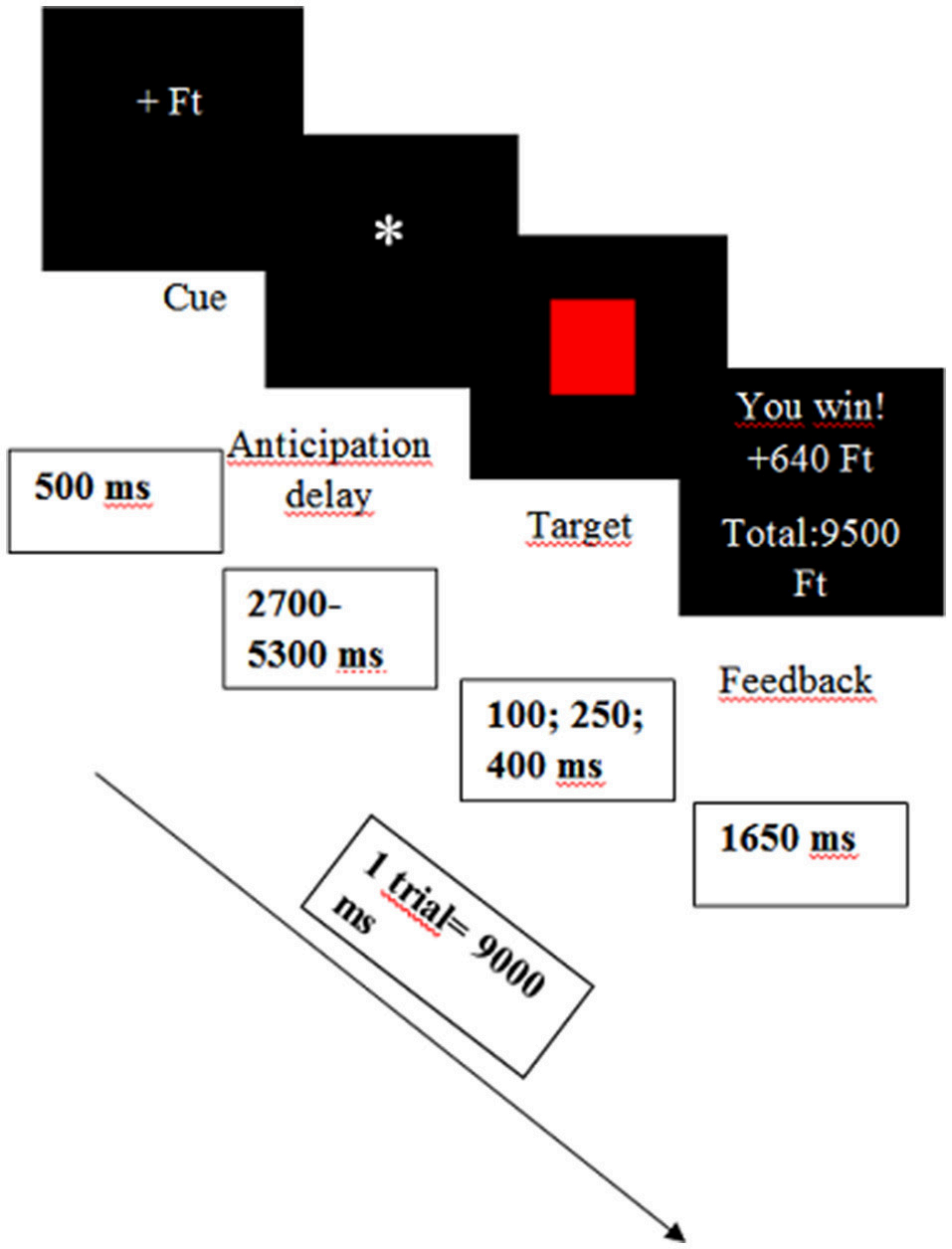
**Design of the Monetary Incentive Delay (MID) task**. Ft, the official abbreviation of the Hungarian currency.

Subjects performed a variant of the classic monetary incentive delay task (MID; Dillon et al., 2008; Pizzagalli et al., 2009), which was designed to evoke neural responses to monetary rewards and losses. During the task, participants could gain money or avoid monetary loss if they responded to a target (a red square) fast enough. The task contained 90 trials organized in two blocks (2 × 45 trials). Each trial consisted of three phases: anticipation, target-response and feedback. During the anticipation phase a visual cue appeared on the screen (for 500 ms) indicating a potentially rewarding (+Ft—which is the official abbreviation of the Hungarian currency), losing (−Ft), or neutral (0 Ft) outcome. Cue presentation was followed by a variable time interval delay (ISI = 2,700–5,300 ms) while a star was presented to the subjects. After the delay a red target square appeared (for 100, 250, or 400 ms), whereupon the participants had to respond with a button press as quickly as possible. Following their response, subjects immediately received feedback (which was visible for 1,650 ms), which informed them whether they won or lost money. Cumulative earnings were also presented during the task (see Figure 1). The trials were separated by a variable inter-trial interval (ranging from 1,150 to 4,050 ms). Each trial lasted 9,000 ms in total. Participants were instructed to respond rapidly in order to maximize their rewards, however the probability of monetary gains or losses was fixed (success index) and was not related to the actual reaction time of the subjects, although, because of credibility, the original script could change if the participants did not respond at all (i.e., in a trial programed to be successful they would lose money). The trials programed to be successful could lead to monetary gains (ranging from 550 to 700 Ft ~ 1.76–2.47€) in the reward condition, or no change in of the penalty condition. The unsuccessful trials also had two outcomes: no change in reward condition and monetary loss in penalty condition (ranging from −550 to 660 Ft ~−1.76 to 2.12€). The success index did not impact considerably the outcome in the no change conditions since the total amount of money stayed unvaried.

The successful-unsuccessful conditions also had influence on the time of the target presentation. In order to increase the credibility, in the successful trials the cues were visible for 400 ms, in the unsuccessful trials the cues were detectable for 100 ms and in the no change condition for 250 ms (for the construction of the task see Table 1).

**Table 1:** **Construction of the monetary incentive delay task**.

**Success index**	**Cue**	**Visibility of the target (ms)**	**Outcome**
Success	+Ft	400	You won
	−Ft	400	No loss
	0 Ft	250	No change
Fail	+Ft	100	No gain
	−Ft	100	You lose
	0 Ft	250	No change

*Ft, the official abbreviation of the Hungarian currency*.

Before entering the scanner, the task was explained to the participants and all of them completed a short practice session (13 trials) on a laptop out of scanner. To maximize engagement, participants were told that after the experiment they would take part in a prize draw to potentially win the money collected during the MID task.

### fMRI acquisition

Functional MRI data acquisition was performed on a 3T MRI scanner (Achieva 3T, Philips Medical System) using a BOLD-sensitive T2^*^-weighted echo-planar imaging sequence (TR = 2,500 ms, TE = 30 ms, FOV: 240 × 240 mm^2^) with 3 × 3 mm in-plane resolution and contiguous 3-mm slices providing whole brain coverage. A series of high-resolution anatomical images were also acquired during the functional imaging session using a T1-weighted 3D TFE sequence with 1 × 1 × 1 mm resolution.

### Statistical analysis

Behavioral data including reaction times in response to the target were recorded using E-prime 2.0 software (Psychology Software Tools, Pittsburgh, PA). Demographic and behavioral data were analyzed in SPSS version 22.0 (IBM SPSS, IBM Corp, Armonk, NY.) using repeated measures ANOVA, *t*-tests and correlation analyses as appropriate. All statistical testing used a two-tailed p < 0.05 threshold.

### fMRI data analysis

Functional imaging data were analyzed with Statistical Parametric Mapping 12 software (http://www.fil.ion.ucl.ac.uk/spm/software/spm12/; Friston et al., 2007). After converting the raw data to NIfTI format, data were pre-processed which included realignment, co-registration to the structural image, segmentation, normalization in Montreal Neurological Institute (MNI) space and spatial smoothing with an 8 mm Gaussian kernel.

For the first level analysis the BOLD (blood oxygenation level-dependent) hemodynamic responses were modeled in a mixed design using the general linear model. Each of the three types of incentive cues (win, loss, neutral) were modeled as blocks, while the five types of feedback (you won, you lost, no gain, no loss, no change) were modeled with event-specific regressors. One additional regressor was defined for motion correction using Artifact Detection Tools (ART; http://www.nitrc.org/projects/artifact_detect/, Whitfield-Gabrieli and Mozes, 2009, MIT).

To capture the brain activation of anticipation, one-sample *t*-tests were performed on three contrasts: reward anticipation was modeled by the win cue-neutral cue [+Ft vs. 0 Ft] contrast, penalty anticipation were assessed by the loss cue-neutral cue [−Ft vs. 0 Ft] contrast and reward anticipation vs. penalty anticipation was modeled by the win cue-loss cue [+Ft vs. −Ft] contrast.

The outcome phase of the task was also modeled by three contrasts: win outcome-neutral outcome, [You won vs. No change], loss outcome-neutral outcome [You lost vs. No change], and win outcome-loss outcome [You won vs. You lost]. Regarding the activations of reward and loss anticipation/consumption a whole brain analysis was carried out at a *p* < 0.01 uncorrected level and cluster level family wise error corrected *p*_FWE_ < 0.05 values were considered as significant with a voxel clustering value of >10.

To investigate the rumination dependent activations associated with reward and loss anticipation [win cue-neutral cue; loss cue-neutral cue; win cue-loss cue] and consumption [win outcome-neutral outcome; loss outcome-neutral outcome; win outcome-loss outcome] whole brain regression analyses were conducted during the second level analysis. Every contrast served as dependent factor and the individual rumination scores were included in the analysis as covariates along with the age, sex, and Zung depression scores. A whole brain analysis were carried out at a *p* < 0.01 uncorrected level and cluster-level family wise error corrected *p*_FWE_ < 0.05 values were reported as significant (with a cluster size >10).

Activated clusters were identified with WFU PickAtlas toolbox, which is based on the Talairach Daemon database (Lancaster et al., 1997, 2000; Maldjian et al., 2003).

## Results

### Self-reported and behavioral results

The mean RRS score was 48.49 (*SD* = 11.55), and the mean ZSDS score was 34.76 (*SD* = 6.52). The ZSDS scores were below the level indicating depression (48 points) and corresponded to the Hungarian healthy average score (34.4 points; Simon, 1998). The correlation between the two scales was significant (Pearson *r* = 0.59; *p* < 0.01). Neither of the two constructs showed any sex differences (RRS: *t* = 0.91 *p* = 0.41; ZSDS: *t* = 0.91 *p* = 0.37).

A repeated measures ANOVA was conducted on the reaction times. A main effect of cue was observed (*F* = 7.19; *p* < 0.05). Subjects responded more quickly both to reward cues (Mean = 238.55 ms; *SD* = 30.10; *p* < 0.001) and to loss cues (Mean = 245.61 ms; *SD* = 30.92; *p* < 0.05) compared to neutral cues (Mean = 262.12 ms; *SD* = 43.82). The cue × RRS interaction was not significant (*F* = 0.35; *p* = 0.97), indicating that reaction times of participants did not vary according to rumination between the three incentive conditions.

### fMRI results

#### Task related activations

The reward anticipation contrast [win cue-neutral cue] showed significant positive activations in four clusters. These clusters covered numerous regions including the occipital lobe and left thalamus. Loss anticipation contrast [loss cue-neutral cue] yielded positive activations in two clusters which covered regions such as the right caudate or left thalamus (for a full overview see Table 2). The reward vs. loss anticipation contrast [win cue-loss cue] revealed no significant brain activation.

**Table 2:** **Peak activity for monetary reward and loss anticipation**.

**Contrast**	**Cluster size (voxels)**	**Region**	**Hemisphere**	**Peak *T*-value**	**Coordinates (MNI)**		
					***x***	***y***	***z***
Win cue-neutral cue	165	Superior occipital gyrus	Right	5.69	21	−82	20
		Superior occipital gyrus	Right	5.21	18	−79	29
		Cuneus	Right	4.04	24	−67	20
	79	Lingual gyrus	Right	5.04	24	−91	−4
		Inferior Occipital Gyrus	Right	4.72	36	−85	−7
		Middle occipital gyrus	Right	4.21	45	−79	−1
	93	Thalamus	Left	4.74	−6	−13	11
		Thalamus	Left	4.59	−3	−16	−1
	176	Middle occipital gyrus	Left	4.54	−21	−82	20
		Superior occipital gyrus	Left	4.28	−21	−67	26
Loss cue-neutral cue	83	Thalamus	Left	4.49	−9	−10	17
		Thalamus	Left	3.88	−6	−16	2
	107	Caudate	Right	4.19	12	−1	17
		Caudate	Right	4.18	9	14	−7

Reward consumption contrast [win outcome-neutral outcome] revealed increased activity in numerous regions for example the anterior cingulate cortex (ACC), right insula, orbitofrontal cortex and superior frontal gyrus, within the eight significant clusters. Loss consumption contrast [loss outcome-neutral outcome] showed positive activations in two clusters covering the regions of the right insula, bilateral lingual gyrus and orbital part of the inferior frontal gyrus (IFG). The reward vs. loss consumption contrast [win outcome-loss outcome] yielded increased activations in multiple regions within nine clusters such as the ACC, right putamen, and right fusiform gyrus (for full list of activations see Table 3).

**Table 3:** **Regions activated in monetary reward and loss consumption**.

**Contrast**	**Cluster size**	**Region**	**Hemisphere**	**Peak *T*-value**	**Coordinates (MNI)**			**BA**
					***x***	***y***	***z***	
Win outcome- neutral outcome	2,983	Lingual gyrus	Left	16.10	−15	−88	−13	
		Middle occipital gyrus	Left	15.55	−12	−94	−1	17
		Fusiform gyrus	Right	15.44	27	−67	−13	
	124	Insula	Right	9.08	39	20	−13	47
		Inferior frontal orbital gyrus	Right	6.63	42	32	−13	47
	224	Superior frontal gyrus	Left	8.19	0	59	8	
		Anterior cingulate	Right	7.08	3	47	14	10
		Anterior cingulate	Right	6.53	3	50	23	9
	67	Superior frontal gyrus	Right	6.52	15	26	56	
		Superior frontal gyrus	Right	6.29	18	32	50	
	32	Angular gyrus	Right	7.02	36	−67	44	7
	21	Middle frontal gyrus	Right	6.63	48	35	17	46
	11	Precuneus	Right	6.15	6	−70	35	
	12	Middle temporal gyrus	Left	6.01	−54	−4	−16	
Loss outcome-neutral outcome	832	Middle occipital gyrus	Left	12.75	−12	−94	−1	17
		Lingual gyrus	Left	12.69	−15	−88	−13	
		Lingual gyrus	Right	10.78	15	−82	−10	
	58	Insula	Right	7.68	33	20	−13	
		Inferior frontal orbital gyrus	Right	6.77	42	20	−19	
Win outcome-loss outcome	498	Inferior occipital gyrus	Left	11.52	−39	−76	−13	
		Middle occipital gyrus	Left	9.16	−27	−82	14	
		Fusiform gyrus	Left	8.15	−33	−43	−22	
	101	Fusiform gyrus	Right	8.19	30	−79	−16	
		Lingual gyrus	Right	7.03	18	−88	−13	
			Right	6.25	6	−85	−10	18
	81	Anterior cingulate	Right	7.11	12	17	−10	
		Putamen	Right	7.00	18	14	−4	
	63	Fusiform gyrus	Right	7.02	33	−43	−19	
	16	Middle frontal gyrus	Right	6.97	33	14	56	8
	18	Precentral gyrus	Left	6.75	−51	8	38	9
	30	Middle occipital gyrus	Right	6.15	30	−85	8	
	21	Caudate	Left	5.92	−12	14	−10	46
	10	Inferior frontal gyrus triangularis	Left	5.88	−45	29	23	

*BA, Brodmann area. Cluster level p_FWE_ < 0.05*.

#### Regression analyses with rumination scores

**Figure 2:**
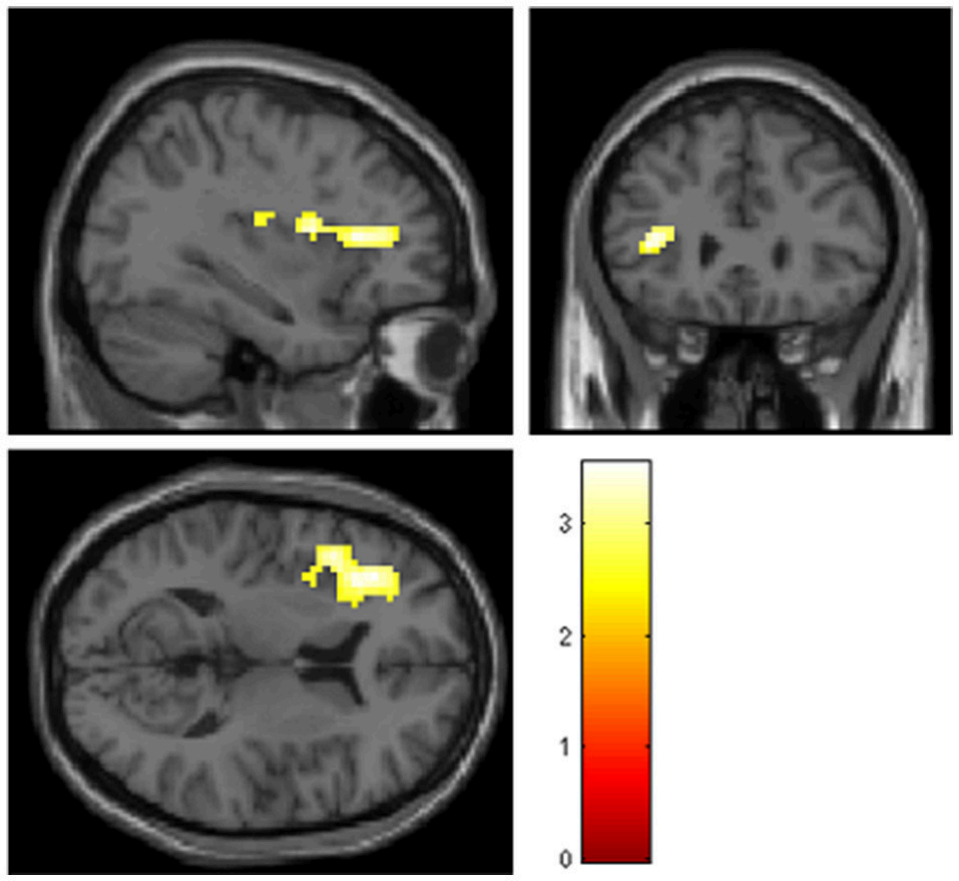
**Significant positive activations (p_**FWE**_ < 0.05, cluster > 10) including peak voxel MNI coordinates, in the left inferior frontal gyrus pars triangularis (***x*** = −36, ***y*** = 32, ***z*** = 11), the left rolandic operculum (***x*** = −42, ***y*** = −19, ***z*** = 20), and the left anterior insula (***x*** = −33, ***y*** = 5, ***z*** = 14) for reward vs. loss anticipation contrast in correlation with rumination scores controlling for age, sex, and ZSDS depression scores**. ZSDS, Zung Self-Rating Depression Scale.

To determine the influence of rumination on the brain activations related to monetary rewards and losses individual rumination scores were entered in the analyses as covariates. The analyses were controlled for age, sex, and ZSDS depression scores. Figure 2 shows the significant activated cluster.

The reward vs. loss anticipation contrast [win cue-loss cue] showed significant positively correlated activations with RRS in one cluster, where the peaks were at the left IFG pars triangularis, the left rolandic operculum and left anterior insula (see Table 4). No other contrast revealed significant brain activations in relation with RRS rumination scores, after FWE corrections.

**Table 4:** **Results of the whole brain regression analysis for reward anticipation vs. loss anticipation contrast [win cue-loss cue] in the relation of rumination, controlled for age, sex, and ZSDS depression scores**.

**Contrast**	**Cluster size**	**Region**	**Hemisphere**	**Peak *T*- value**	**Coordinates (MNI)**		
					***x***	***y***	***z***
Win cue- loss cue	311	Inferior frontal gyrus triangularis	Left	3.55	−36	32	11
		Rolandic operculum	Left	3.34	−42	−19	20
		Anterior insula	Left	3.33	−33	5	14

*Cluster level p_FWE_ < 0.05*.

## Discussion

In our study, we explored the neural correlates of trait rumination during reward and loss anticipation and consumption measured by a variant of the MID task, in a healthy, never-depressed sample. We found significant positive correlation between rumination and reward anticipation (win cues) compared to loss anticipation (loss cues). More precisely, trait rumination -controlled for age, sex, and depressed mood-correlated with one significant cluster of 311 voxels, where the peaks were at the left anterior insula, left inferior frontal gyrus pars triangularis (IFG), and left rolandic operculum. We did not detect any significant rumination-related activations yielded for win-neutral and loss-neutral cues, and reward or loss consumption (win-loss outcome).

Our results might indicate elevated sensitivity to reward cues among ruminators, since during reward anticipation they tended to more actively recruit brain areas together, such as anterior insula (AI) and IFG, which are implicated in the salience network (SN; Wiech et al., 2010; Chang et al., 2013; Menon, 2015).

AI is involved in the processing of multimodal stimuli, since it receives input from multiple brain areas such as the amygdala, ventral tegmental area, or ventral striatum (Cauda et al., 2012). Convergent evidence suggests, that the AI, as the final location of hierarchical information processing, could provide access to the reward or punishment saliency of stimuli (Pizzagalli et al., 2009; Critchley et al., 2013), which could also explain the consistently found hyperactivity of AI in mood and anxiety disorders (Paulus and Stein, 2006; Hamilton et al., 2013).

It is still unclear however, whether the anticipation or consumption phase of the processing is linked to heightened AI (and SN) response, and whether reward or punishment (loss) signals are more important for ruminators.

Before we interpret the role of rumination on this matter, it is worthwhile to discuss the neuronal activity involved in reward/punishment processing in general. Evidence from animal studies (Berridge and Robinson, 1998; Haber and Knutson, 2010) suggests that there is a neural network, which is highly sensitive to the rewarding nature of stimuli. The cortical-basal ganglia circuit is the cornerstone of the reward system, specifically the ventral striatum (along with the nucleus accumbens), orbitofrontal cortex, ACC, and the midbrain dopamine neurons (Haber and Knutson, 2010). However, the non-human primates' studies (Schultz et al., 2000) demonstrated that the activity of these key structures was dependent on the temporal phases of reward/punishment processing. The ventral striatal regions and orbitofrontal regions are activated during the expectation (or “wanting”) period preceding reward, while the medial and ventromedial prefrontal cortex show greater activity during the receipt of reward (or during the “liking” phase; Lutz and Widmer, 2014; Kaskan et al., 2017). Although the orbital part of the frontal cortex is the region most often associated with reward in animal studies, the significance of the ventrolateral prefrontal cortex (vlPFC) was also supported, especially in value encoding and reward-guided decision making (Kaskan et al., 2017).

In line with these animal studies, several human findings linked the anticipation phase to motivational processes, which foster goal-directed behavior targeting the desired outcomes, while the consumption phase is associated with hedonic processes, where the focus is on the experience of the pleasurable state (Gard et al., 2006; Dillon et al., 2008). Over the past decades, many human studies have used the MID task to test empirically this theoretical dissociation. Like animal studies most of them found that the wanting and liking phases are mediated (at least partially) by separable neural systems (Knutson et al., 2001, 2003).

Our findings referring to the task related activations, seem to concord with the above mentioned results, since striatal regions were activated during anticipation and frontal areas (such as vlPFC) yielded activations for consumption. However, when inter-individual differences such as depressive mood or rumination were also modeled in our fMRI analyses, AI yielded positive activation for reward anticipation, but not for the consumption phase of reward processing. Our results are in line with the results of Strigo et al. (2008) who investigated the pain processing of MDD patients and compared them to a control group; they found increased activation in AI during the anticipation but not the experience of pain stimuli (Strigo et al., 2008). In addition, Wiech et al. (2010) were able to detect heightened AI activation in healthy participants during anticipation of pain (Wiech et al., 2010), however, there are conflicting results too (Craig, 2009). Studies with (remitted) depressed patients have found elevated AI activation during the consumption and not the anticipation of negative stimuli (Craig, 2009; Hamilton et al., 2013).

According to some previous results (Strigo et al., 2008; Pizzagalli et al., 2009) increased SN activity associated with rumination was detected only for negatively-valenced stimuli (e.g., for monetary loss, pain), however, it is important to note that the participants in those studies were depressed or remitted depressed patients. For this reason, it is not clear whether these findings are connected directly to rumination or could be attributed to current or past depressed mood.

Besides the AI, we found rumination related peak activation in the left IFG pars triangularis during reward anticipation. The pars triangularis of the IFG, or more broadly the ventrolateral prefrontal cortex (vlPFC; Ridderinkhof et al., 2004) has a central role in inhibitory control (Swick et al., 2011), specifically in the inhibition of irrelevant or negative information from short-term memory (Berman et al., 2011), and has an important function in memory retrieval (Badre and Wagner, 2007). This brain area is among the most frequently activated regions in connection with rumination (Piguet et al., 2014). These findings are also convergent with the results of Kühn et al. (2013), who detected greater left IFG activations among those non-depressed people who tend to experience intrusive thoughts (Kühn et al., 2013). Our results are consistent with previous fMRI studies, which demonstrated that, beyond its role in the control and memory processes, lPFC also mediates prediction of reward information (Dixon and Christoff, 2014). Tanaka et al. (2015) also found that lPFC neurons play a role in both abstract categorization and stimulus—reward associations (Tanaka et al., 2015).

In addition, we detected heightened neural response during reward anticipation in a third peak, in the rolandic operculum (RO). Although, the exact role of RO in rumination is unclear, evidence suggests that increased RO activation is correlated with some anticipatory processes. For instance, obese adolescents showed greater activations in RO during anticipation of food reward than thin counterparts (Stice et al., 2008).

All of these activations were yielded for win-loss anticipation in connection with rumination. However, when we analyzed the main effect of the win-loss cue contrast, we did not get significant results. It can be argued that the task was not robust enough referring to win-loss anticipation, but we would like to emphasize that this contrast is not standard in the literature. Most of the works using MID task (Knutson et al., 2001, 2003; Pizzagalli et al., 2009) only model the win-neutral cue or loss-neutral cue contrasts, which are designed to capture the reward or loss related activity, controlling for sensory processing and motor preparation. Nonetheless, these contrasts do not control for those anticipatory processes or for the emotional arousal, which could be evoked by the expectations of *both* rewards and losses. For this reason, the reliable distinction of areas, which are involved exclusively in reward anticipation or just in loss anticipation, is questionable (Dillon et al., 2008). We intended to bridge this gap; therefore, we incorporated the win-loss cue contrast into our analysis.

There are previous findings (Cooper and Knutson, 2008), suggesting that the valence and discriminative power of the anticipatory stimuli (i.e., the cues), may not be relevant in certain cases especially when the rewards are unexpected or unpredictable. Since in our study several activations yielded for contrasts where the valence of the stimuli is obvious and the distinctive power of the cues is smaller (namely the win-neutral cue and loss-neutral cue contrasts), the non-significant result for the win-loss cue could be the outcome of the unpredictable nature of the MID task. In addition, when we tested the win-loss cue contrast without covariates, we did not investigate the effects of the individual differences, but when all of the covariates were entered in the model, these important differences manifested. As we have outlined in the introduction, the individual characteristics could play an important role in information processing (Whitmer et al., 2012; Whitmer and Gotlib, 2013). We hypothesized that rumination could be one of these influential characteristics.

Our results suggest that for never-depressed ruminators, potentially rewarding cues may be more salient than loss cues. One interpretation of our findings could be, as we have outlined in the introduction, that rumination, as an abstract information processing mode, leads to over-simplified representations and thus impaired anticipation of the future events (Watkins et al., 2015). This suggests that rumination is more likely to correlate with the anticipatory but not the consumption phase of processing. Ruminators tend to exaggerate the importance of negative information and overgeneralize and amplify the incidence of casual failures (Van Lier et al., 2014) and according to our results trait rumination might be associated with enhanced reward expectations. However, further investigations are needed to clarify the complex association between rumination and reward anticipation.

### Limitations

Some limitations of the study should be taken into consideration. Since we focused our investigation on trait level rumination rather than active, current rumination we used the total RRS scores, and we did not induce or measure state rumination. Furthermore, we did not measure explicitly the motivation to win or avoid loss, although from the behavioral results (faster reaction times to win/loss cues than neutral cues) we can conclude that participants were indeed motivated. In addition, we are aware that we used a liberal primary threshold during the analysis which can be arguable, but since this is an exploratory study and the significant activated cluster was not too large we think this was a valid decision. Finally, since few studies have previously investigated the neural background of rumination and reward/loss processing (Dichter et al., 2012; Schiller et al., 2013), particularly in healthy, never- depressed participants, the comparability or generalization of our results is limited.

## Conclusion

To the best of our knowledge, this is the first neuroimaging study that has investigated the impact of trait rumination, independent of depressed mood, on reward and punishment (loss) anticipation and consumption in healthy participants. Our results suggest that trait rumination has a significant influence on the anticipation but not on the consumption of rewards. These results might suggest that rumination alters processing of the motivational (wanting) aspect but not the hedonic (liking) aspect of monetary reward, at least in the absence of pathological mood. Further studies will be needed to investigate how rumination affects reward-related prediction error, when there is a difference between the expected and the experienced reward.

## Author contributions

GJ and GK conceived and designed the study. NK, ES, AÉ, AG, and DP were responsible for subject recruitment and data collection. Data analysis were performed by NK and GK with special assistance from LK and GJ. RE, GB, and GJ contributed to the interpretation to the data. NK, GK wrote the manuscript and all authors provided critical revision to its further development. All authors read and approved the final manuscript.

## Funding

The author(s) disclosed receipt of the following financial support for the research, authorship, and/or publication of this article: the study was supported by the MTA-SE-NAP B Genetic Brain Imaging Migraine Research Group, Hungarian Academy of Sciences, Semmelweis University (Grant No. KTIA_NAP_13-2-2015-0001); and the Hungarian Academy of Sciences (MTA-SE Neuropsychopharmacology and Neurochemistry Research Group). LK was supported by the Bolyai Research Fellowship Program of the Hungarian Academy of Sciences. RE received consultancy fees from Cambridge Cognition and P1 vital.

### Conflict of interest statement

The authors declare that the research was conducted in the absence of any commercial or financial relationships that could be construed as a potential conflict of interest.
